# Study on mechanical characteristics of anchor drilling rig group for rapid excavating

**DOI:** 10.1038/s41598-023-31809-z

**Published:** 2023-03-20

**Authors:** Miao Xie, Hong-yu Zhang, Yu-qi Li, Ze Ren, Ren-dong Nie

**Affiliations:** 1 College of Mechanical Engineering, Liaoning Technical University, Fuxin, Liaoling China; 2 National Energy Group International Engineering Consulting Co Ltd, Beijing, China; 3 College of Mining, Liaoning Technical University, Fuxin, Liaoling China

**Keywords:** Mechanical engineering, Applied mathematics, Computational science

## Abstract

Aiming at the imbalance problem of excavating and anchoring efficiency ratio in the underground coal mine, a new type of parallel operation unit of excavation-supporting-anchoring is proposed to improve the excavating efficiency. Considering the uneven factors of the roof and floor of the coal mine roadway, the influence of different drilling angles and leg support angles on the leg support force is studied under the condition of simultaneous drilling of multiple drilling rigs. The results show that the maximum support force is 4.002 KN when the leg angle is different and the drilling angle is different. By constructing the coupling vibration model of the multi-drill drilling process and using the Lagrange method to solve the vibration law of key components of the anchorage system, the vibration law of drill pipe under different influencing factors such as cantilever extension, multi-drill simultaneous drilling, and drilling incident angle is studied. The results showed: (1) The vibration of the drill pipe in the cantilever extension state is more severe than that in the retracted state, and the maximum vibration peak reaches 7.61 mm. (2) The vibration response of the drill pipe is the most intense under the condition of four top anchor drilling rigs drilling at the same time. Under the working condition of only two drilling rigs, the vibration response of the drill pipe is the smallest. (3) As the drilling angle of the drilling rig increases, the vibration response of the drill pipe is more severe and the vibration amplitude is larger. A test prototype is built to simulate the actual anchoring drilling process, and the vibration law of the support platform and the drilling rig is obtained through the vibration detection system. The test results show that the vibration law of the key components is approximately the same as the theoretical simulation results. The relevant theoretical results can provide a technical basis for the drilling stability of the anchoring system.

## Introduction

The proportion of underground mining is seriously out of balance, and the level of mining automation is relatively backward. The disadvantages of a long time and high strength of anchoring operation seriously restrict the development of excavation operation. Foresight Energy has designed a 6-arm anchor rod drill truck, which can synchronously realize roof anchoring operation and improve anchoring efficiency^1^.

During drilling, uncontrollable vibration often occurs, resulting in low drilling efficiency and increased cost^2^. The existence of special excitation during drilling usually leads to uncertain vibration response^3–6^. The works^9–12^ studied the discontinuous vibration behavior of periodic motion based on a 3-dof collision system. In Table 1, the dynamic response and vibration control method strategy in the multi-degree-of-freedom vibration system are proposed, which improves the system response performance.

**Table 1:** Vibration impact system analysis.

Author	References
Analyzed the dynamic response of vibration through the Poincare diagram based on the multi-degree-of-freedom vibration impact system	Luo et al.^7^
The symmetry of the Poincare map significantly affects the bifurcation behavior of the vibration impact system, which not only suppresses the period-doubling bifurcation, but also suppresses the Hopf-flip bifurcation and pitchfork-flip bifurcation	Yue et al.^8^
The vibration of the liquid storage tank is studied, and the corresponding tractor is established. The bifurcation is avoided by displacement feedback control to achieve the purpose of vibration control and improve the response performance of the vibration impact system	Liu et al.^13^

Considering the interaction between bit and rock vibration, relevant scholars have established a system model of the drilling process to study the axial vibration law of drill string under coupling or uncoupling action^14,15^. The work^16^ analyzed the axial vibration law of drill pipe with the partial differential equation of drill string. In work^17^ studied the mathematical model of axial vibration of drill string under combined excitation. Investigations of work^18^ studied the interaction between the drill string and wellbore during tripping out by building a static analysis model. The work^19^ established a vibration analysis model based on plane wave propagation through the distributed mass of drill string. Yigit and Christofru^21^ studied the axial load and transverse vibration load law of the drill string by simplifying the drill string model as a slender beam. In Table 2, the multi-directional vibration response law of drill string in the drilling process is studied by using finite element and virtual simulation technology.

**Table 2:** Vibration characteristics analysis of drill string drilling process.

Author	References
By establishing a finite element mechanical model, the dynamic response of the drill string under impact can be further predicted	Kalsi et al.^20^
The lateral and vibration response laws of the drill pipe during the top anchor drilling process are obtained by virtual simulation technology	Fu et al.^22^

Given the research on the performance prediction of anchors by NDT, the work^23^ quantifies the crack risk by temperature stress analysis to improve the accuracy of the machine by compressing the data used. The experimental research work in this work^24^ introduces in detail the development of a non-destructive testing method, which estimates the crack condition of concrete around steel bars by ultrasonic pulse velocity test. The work^25^ presents the first true non-destructive testing method using the rebound number of Schmidt hammers to estimate the bearing capacity of bolts. The work^26^ presented in this paper details a true non-destructive testing program that can be used to evaluate the pull-out strength of concrete anchors.

In this paper, the anchoring group drilling rig is taken as the research object, and the static and dynamic laws of the anchoring system under the complex working conditions of the roof and floor of the roadway are studied. The experimental prototype is used to simulate the drilling test, and the actual vibration law of the key components of the anchoring system is obtained. The relevant theoretical results in this paper can provide a technical basis for the drilling stability of the anchoring system.

## Composition of the excavation-support-bolting combined unit

**Figure 1:**
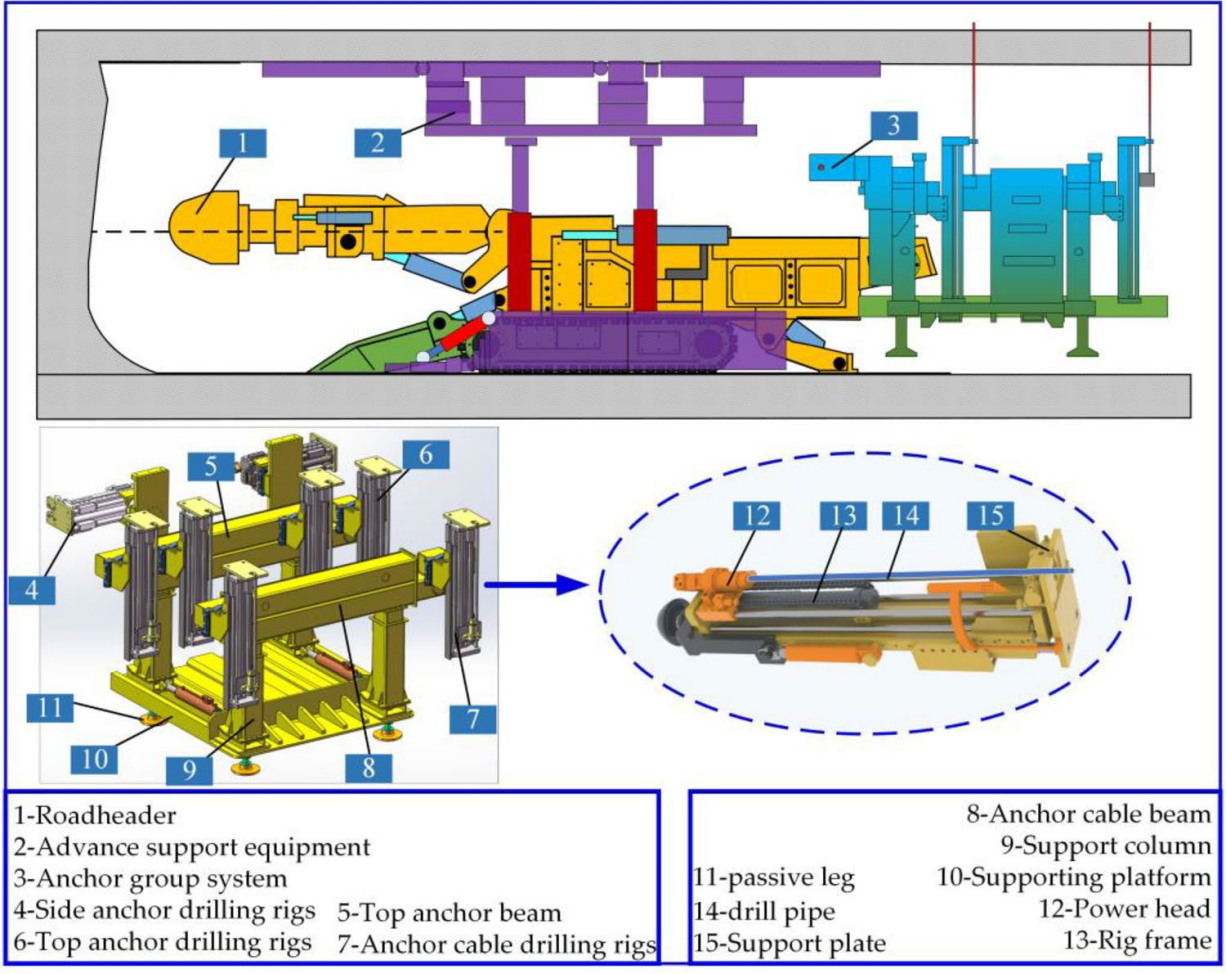
Equipment composition diagram of driving, supporting and anchoring combined unit.

The existing rapid excavation system has a poor practical application effect in coal mines. There are mainly problems such as poor stability of the fuselage, low efficiency, and difficulty in moving with the machine during anchoring operation. Because of the existing problems of parallel operation of excavation and anchoring in the above-mentioned fully mechanized excavation face, this paper proposes a new type of rapid excavation unit for parallel operation of excavation, support, anchoring, and transportation. It is mainly composed of four parts: roadheader, advanced support equipment, anchoring group system, and transportation system, as shown in Fig. 1. The advanced support equipment adopts the step-by-step non-repetitive rolling method, and there are multiple sets of flexible support units above the support, which can adapt to the temporary support operation under different geological conditions. The excavating system and the anchoring system can realize walking synchronization and operation separation. As an important part of the new intelligent excavating system, the anchoring system is of great significance to the study of its mechanical properties and reliability.

## Construction and solution of anchorage group statics model

**Figure 2:**
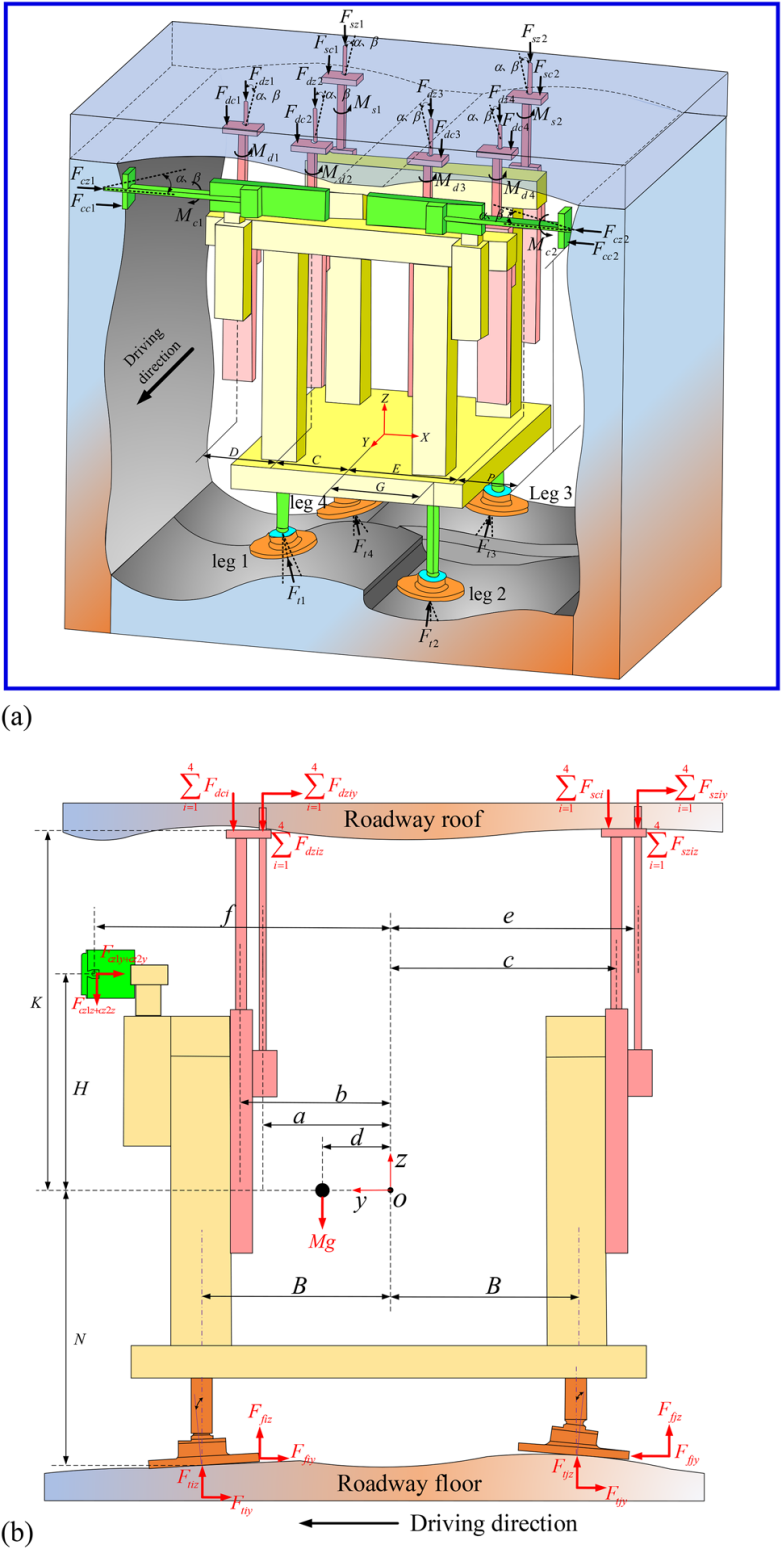
Mechanical model of anchorage group under overall uneven working condition of roadway floor: (**a**) axonometric drawing of anchorage system; (**b**) side view of anchorage system.

Considering the complex conditions of the roadway roof and floor, the static model of the anchorage group is constructed, as shown in Fig. 2. Among them,* F*_*t1*_ ~ *F*_*t4*_ is the four-leg support force, *F*_*f1*_ ~ *F*_*f4*_ is the four-leg friction force, *F*_*cc1*_ ~ *F*_*cc2*_ is the side anchor support reaction force, *F*_*cz1*_ ~ *F*_*cz2*_ is the side anchor drilling reaction force, *F*_*dc1*_ ~ *F*_*dc4*_ is the top anchor support reaction force, *F*_*dz1*_ ~ *F*_*dz4*_ is the top anchor drilling reaction force, *F*_*sc1*_ ~ *F*_*sc4*_ is the anchor cable support reaction force, *F*_*sz1*_ ~ *F*_*sz4*_ is the anchor cable drilling reaction force. Set the *O* point as the center position of the system, and establish the coordinate system *OXYZ* as shown in Fig. 2a. The top anchor drilling rigs, side anchor drilling rigs, and anchor cable drilling rigs are symmetrically arranged in the *YZ* section. The distance between the top anchor drilling rig and the *YZ* section is *C*, the distance between the inner side and the outer top anchor drilling rig is *D*, the distance between the supporting column and the *YZ* section is *G*, the distance between the anchor cable drilling rig and the *YZ* section is *E*, and the distance between the anchor cable drilling rig and the side coal wall is* P*. The projection of the *YZ* section is shown in Fig. 2b. The distance from the center of gravity of the system to the *XZ* section is *d*, the distance from the center of the top anchor support plate to the *XZ* section is *b*, the distance from the center of the top anchor drill pipe to the *XZ* section is *a*, the distance from the center of the side anchor drill pipe to the *XZ* section is *f*, the distance from the center of the anchor cable drill pipe to the *XZ* section is *e*, the distance from the center of the anchor cable support plate to the *XZ* section is* c*, the distance from the center of the front and rear support columns to the *XZ* section is* B*, the distance from the roadway roof to the *XY* section is *K*, the distance from the center of the top anchor drilling rig to the *XY* section is *H*, the distance from the roadway floor to the *XY* section is* N*, and the weight of the whole machine is *Mg*.

Considering the uneven characteristics of the coal mine roadway floor, the contact relationship between the legs and the roadway floor has a variety of corresponding relationships. The force analysis process of the four legs is similar. The form of the leg under complex working conditions is divided into two cases: inward leg and outward leg.

**Figure 3:**
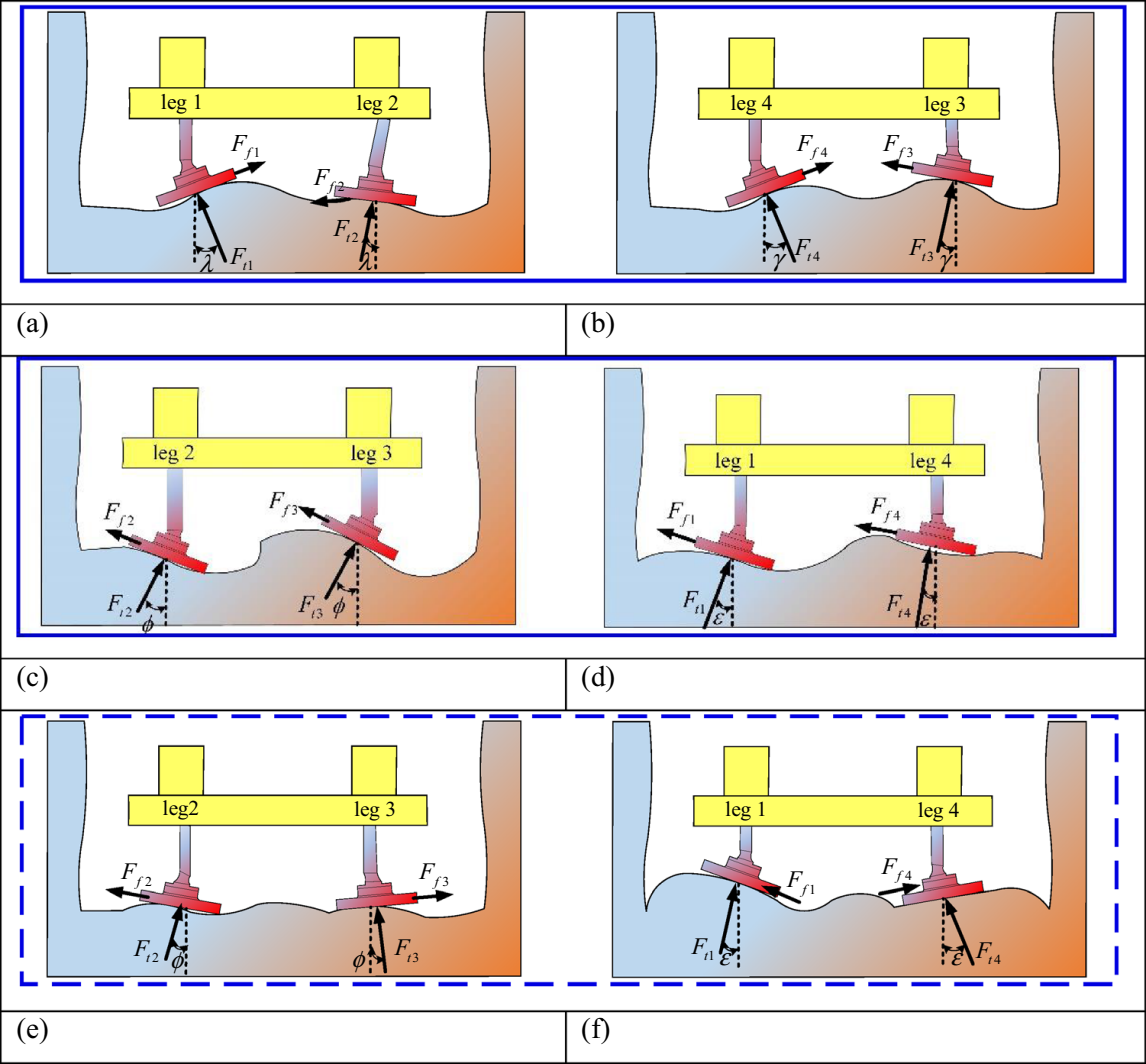
Angle between reaction force of supporting leg and coordinate axis: (**a**) angle between 1 and 2 leg and XZ cross section; (**b**) angle between 3 and 4 leg and XZ cross section; (**c**) angle between 2 and 3 leg and YZ cross section(outward legs); (**d**) angle between 1 and 4 leg and YZ cross section(outward legs); (**e**) angle between 2 and 3 leg and YZ cross section(inward legs); (**f**) angle between 1 and 4 leg and YZ cross section(inward legs).

Outward legs are shown in Fig. 3a–d. It can be assumed that the angle between the support force of the leg 1 and 2 and the ground is $\lambda$ in the *Z–X* coordinate system, and the direction is opposite. In the *Z–X* coordinate system, the angle between the support force of the leg 3 and 4 and the ground is $\gamma$, and the direction is opposite. In the *Z–Y* coordinate system, the angle between the support force of the leg 2 and 3 and the ground is $\phi$, and the direction is the same; In the *Z-Y* coordinate system, the angle between the leg 1 and 4 supporting force and the ground is $\varepsilon$, and the direction is the same.

Inward legs are shown in Fig. 3a, b, e, f. In the *Z–Y* coordinate system, the angle between the support force of the leg 2 and 3 and the ground is $\phi$, and the direction is opposite; In the *Z–Y* coordinate system, the angle between the leg 1 and 4 supporting force and the ground is $\varepsilon$, and the direction is opposite.

**Figure 4:**
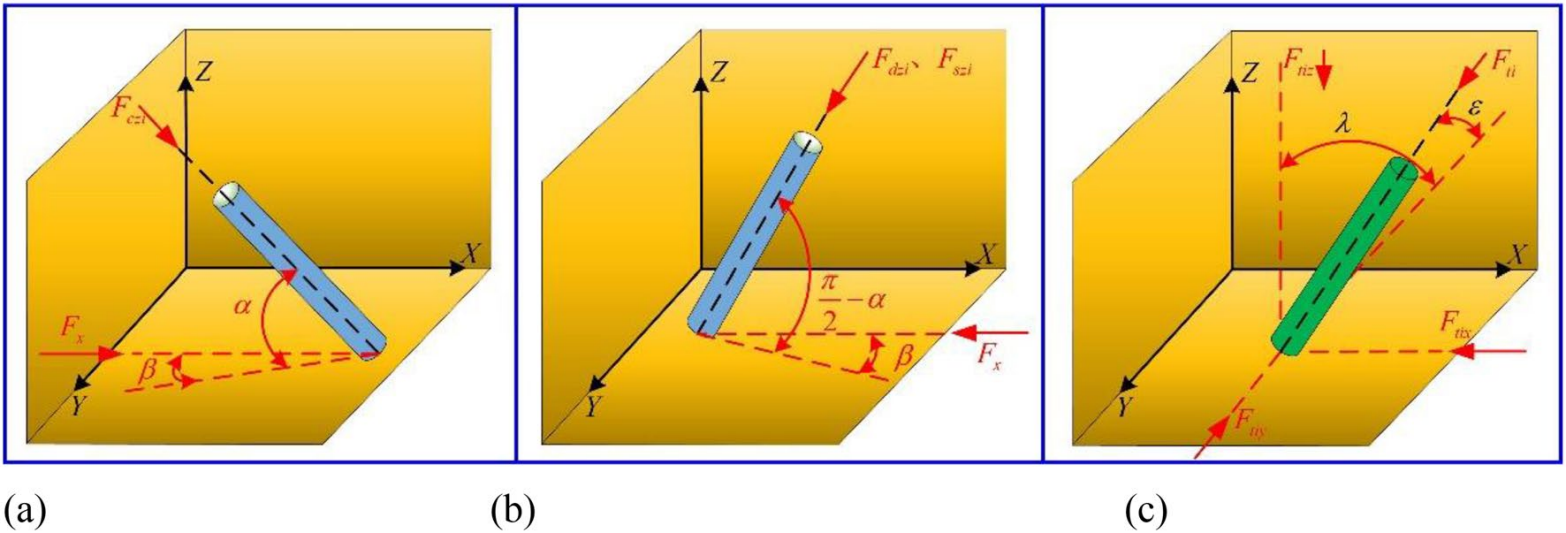
Diagram of relationship between drilling reaction force, leg supporting force and coordinate axis angle: (**a**) relationship between side wall drilling reaction and dip angle; (**b**) relation between top drilling reaction and dip angle; (**c**) schematic diagram of leg reaction and inclination.

By using the automatic adjustment function of the four legs, the anchoring platform can be in a complete horizontal state. But the roadway roof is uneven, and the size and direction of drilling three-way force are uncertain. To study the influence of different incident angles on leg force in the drilling process, the drilling process of multiple drilling rigs can be simplified as the superposition of a single drilling rig under complex working conditions. The two-direction incident angles of the top anchor, side anchor and anchor cable drilling rig are assumed to be $\alpha$ or $\pi /2 - \alpha$ and $\beta$, as shown in Fig. 4a–c. *F*_*cz1x*/*y*/*z*_ ~ *F*_*cz2x/y/z*_ are the component forces of two side-anchored drilling rigs along *x*, *y*, and *z* axes, *F*_*dz1x/y/z*_ ~ *F*_*dz4x/y/z*_ are the component forces of four top-anchored drilling rigs along *x*, *y* and *z* axes, *F*_*sz1x/y/z*_ ~ *F*_*sz2x/y/z*_ are the component forces of two anchor drilling rigs along *x*, *y* and *z* axes, *F*_*t1x/y/z*_ ~ *F*_*t4x/y/z*_ are the component forces of four legs along *x*, *y* and *z* axes, *F*_*f1x/y/z*_ ~ *F*_*f4x/y/z*_ are the component forces of four legs along *x*, *y* and *z* axes.

According to the principle of static force balance:

1 $$ \left\{ \begin{gathered} F_{t1x} = F_{t1} \sin \lambda \cos \varepsilon \hfill \\ F_{t1y} = F_{t1} \sin \varepsilon \hfill \\ F_{t1z} = F_{t1} \cos \lambda \cos \varepsilon \hfill \\ F_{t2x} = F_{t2} \sin \lambda \cos \phi \hfill \\ F_{t2y} = F_{t2} \sin \phi \hfill \\ F_{t2z} = F_{t2} \cos \lambda \cos \phi \hfill \\ Outward \, legs: \hfill \\ F_{t3x} = F_{t3} \sin \gamma \cos \phi \hfill \\ F_{t3y} = F_{t3} \sin \phi \hfill \\ F_{t3z} = F_{t3} \cos \gamma \cos \phi \hfill \\ F_{t4x} = F_{t4} \sin \gamma \cos \varepsilon \hfill \\ F_{t4y} = F_{t4} \sin \varepsilon \hfill \\ F_{t4z} = F_{t4} \cos \gamma \cos \varepsilon \hfill \\ Inward \, legs: \hfill \\ F_{t3x} = - F_{t3} \sin \gamma \cos \phi \hfill \\ F_{t3y} = - F_{t3} \sin \phi \hfill \\ F_{t3z} = - F_{t3} \cos \gamma \cos \phi \hfill \\ F_{t4x} = - F_{t4} \sin \gamma \cos \varepsilon \hfill \\ F_{t4y} = - F_{t4} \sin \varepsilon \hfill \\ F_{t4z} = - F_{t4} \cos \gamma \cos \varepsilon \hfill \\ \end{gathered} \right.\;\;\;\left\{ \begin{gathered} F_{f1x} = \mu F_{t1} \cos \lambda \hfill \\ F_{f1y} = \mu F_{t1} \cos \varepsilon \hfill \\ F_{f1z} = - \mu F_{t1} \sin \lambda - \mu F_{t1} \sin \varepsilon \hfill \\ F_{f2x} = \mu F_{t2} \cos \lambda \hfill \\ F_{f2y} = \mu F_{t2} \cos \phi \hfill \\ F_{f2z} = - \mu F_{t2} \sin \lambda - \mu F_{t2} \sin \phi \hfill \\ Outward \, legs: \hfill \\ F_{f3x} = \mu F_{t3} \cos \gamma \hfill \\ F_{f3y} = \mu F_{t3} \cos \phi \hfill \\ F_{f3z} = - \mu F_{t3} \sin \gamma - \mu F_{t3} \sin \phi \hfill \\ F_{f4x} = \mu F_{t4} \cos \gamma \hfill \\ F_{f4y} = \mu F_{t4} \cos \varepsilon \hfill \\ F_{f4z} = - \mu F_{t4} \sin \gamma - \mu F_{t4} \sin \varepsilon \hfill \\ Inward \, legs: \hfill \\ F_{f3x} = - \mu F_{t3} \cos \gamma \hfill \\ F_{f3y} = - \mu F_{t3} \cos \phi \hfill \\ F_{f3z} = \mu F_{t3} \sin \gamma + \mu F_{t3} \sin \phi \hfill \\ F_{f4x} = - \mu F_{t4} \cos \gamma \hfill \\ F_{f4y} = - \mu F_{t4} \cos \varepsilon \hfill \\ F_{f4z} = \mu F_{t4} \sin \gamma + \mu F_{t4} \sin \varepsilon \hfill \\ \end{gathered} \right.\;\;\;\left\{ \begin{gathered} F_{czix} = F_{czi} \cos \alpha \cos \beta \hfill \\ F_{cziy} = F_{czi} \cos \alpha \sin \beta \hfill \\ F_{cziz} = F_{czi} \sin \alpha \hfill \\ F_{dzix} = F_{dzi} \cos \left( {\pi /2 - \alpha } \right)\cos \beta \hfill \\ F_{dziy} = F_{dzi} \cos \left( {\pi /2 - \alpha } \right)\sin \beta \hfill \\ F_{dziz} = F_{dzi} \sin \left( {\pi /2 - \alpha } \right) \hfill \\ F_{szix} = F_{szi} \cos \left( {\pi /2 - \alpha } \right)\cos \beta \hfill \\ F_{sziy} = F_{szi} \cos \left( {\pi /2 - \alpha } \right)\sin \beta \hfill \\ F_{sziz} = F_{szi} \sin \left( {\pi /2 - \alpha } \right) \hfill \\ \hfill \\ \hfill \\ \hfill \\ \hfill \\ \hfill \\ \hfill \\ \hfill \\ \hfill \\ \hfill \\ \hfill \\ \hfill \\ \end{gathered} \right. $$

2 $$ \left\{ \begin{gathered} \sum {F_{x} } = 0 \hfill \\ - \sin \lambda \left( {F_{t1} \cos \varepsilon + F_{t2} \cos \phi } \right) + \sin \gamma \left( {F_{t3} \cos \phi - F_{t4} \cos \varepsilon } \right) + \left( {F_{f1x} - F_{f2x} } \right) + \left( {F_{f4x} - F_{f3x} } \right) + F_{cc1} \hfill \\ - F_{cc2} + \left( {F_{cz1} - F_{cz2} } \right)\cos \alpha \cos \beta - \sum\limits_{i = 1}^{4} {F_{dzi} } \cos \left( {\frac{\pi }{2} - \alpha } \right)\cos \beta - \sum\limits_{i = 1}^{2} {F_{szi} } \cos \left( {\frac{\pi }{2} - \alpha } \right)\cos \beta = 0 \hfill \\ \end{gathered} \right. $$

3 $$ \left\{ \begin{gathered} \sum {F_{y} } = 0 \hfill \\ - \sin \phi (F_{t2} + F_{t3} ) - \sin \varepsilon (F_{t1} + F_{t4} ){ + }(F_{f2y} + F_{f3y} ) + (F_{f1y} + F_{f4y} ) \hfill \\ - \left( {F_{cz1} + F_{cz2} } \right)\cos \alpha \sin \beta - \sum\limits_{i = 1}^{4} {F_{dzi} \cos \left( {\frac{\pi }{2} - \alpha } \right)} \sin \beta - \sum\limits_{i = 1}^{2} {F_{szi} } \cos \left( {\frac{\pi }{2} - \alpha } \right)\sin \beta = 0 \hfill \\ \end{gathered} \right. $$

4 $$ \left\{ \begin{gathered} \sum {F_{z} } = 0 \hfill \\ \left( {F_{t1} \cos \lambda \cos \varepsilon + F_{t2} \cos \lambda \cos \phi + F_{t3} \cos \gamma \cos \phi + F_{t4} \cos \gamma \cos \varepsilon } \right) - Mg \hfill \\ - \left( {F_{cz1} + F_{cz2} } \right)\cos \alpha - \sum\limits_{i = 1}^{4} {F_{dzi} } \cos \left( {\frac{\pi }{2} - \alpha } \right) - \sum\limits_{i = 1}^{4} {F_{szi} } \cos \left( {\frac{\pi }{2} - \alpha } \right) - \sum\limits_{i = 1}^{4} {F_{dci} } - \sum\limits_{i = 1}^{4} {F_{sci} } = 0 \hfill \\ \end{gathered} \right. $$

5 $$ \left\{ \begin{gathered} \sum {M_{x} } = 0 \hfill \\ \left( {F_{t1y} + F_{t2y} - F_{t3y} - F_{t4y} } \right)N + \left( {F_{t3z} + F_{t4z} - F_{t1z} - F_{t2z} } \right)B + Mgd - \left( {F_{cz1y} + F_{cz2y} } \right)H \hfill \\ + \left( {F_{cz1z} + F_{cz2z} } \right)f - \sum\limits_{i = 1}^{4} {F_{dci} } b + \sum\limits_{i = 1}^{4} {F_{dziy} } k - \sum\limits_{i = 1}^{4} {F_{dziz} } a + \sum\limits_{i = 1}^{4} {F_{sci} } c + \sum\limits_{i = 1}^{4} {F_{sziy} } k + \sum\limits_{i = 1}^{4} {F_{sziz} } e = 0 \hfill \\ \end{gathered} \right. $$

6 $$ \left\{ \begin{gathered} \sum {M_{y} } = 0 \hfill \\ \left( {F_{t3x} - F_{t2x} - F_{t1x} - F_{t4x} + F_{f1x} + F_{f4x} - F_{f2x} - F_{f3x} } \right)N - \left( {F_{cc1} - F_{cc2} + F_{cz1x} - F_{cz2x} } \right)H \hfill \\ - \left( {F_{cz2z} - F_{cz1z} } \right)(P + E){ + }\sum\limits_{i = 1}^{4} {F_{dzix} } K + \left( {F_{dc1} + F_{dz1z} } \right)(D + C){ + }\left( {F_{dc2} + F_{dz2z} - F_{dc3} - F_{dz3z} } \right)C \hfill \\ - \left( {F_{dc4} + F_{dz4z} } \right)(D + C) + \left( {F_{sc1} + F_{sz1z} - F_{sc2} - F_{sz2z} } \right)E + \sum\limits_{i = 1}^{2} {F_{szix} } K = 0 \hfill \\ \end{gathered} \right. $$

7 $$ \left\{ \begin{gathered} \sum {M_{z} } = 0 \hfill \\ \left( {F_{f1x} + F_{f2x} - F_{t1x} - F_{t2x} + F_{f3x} - F_{f4x} + F_{t3x} - F_{t4x} } \right)B + \left( {F_{f1y} + F_{f4y} + F_{t4y} - F_{t1y} - F_{t2y} } \right. \hfill \\ + F\left. {_{t3y} + F_{f2y} + F_{f3y} } \right)G - \left( {F_{cc2} - F_{cc1} - F_{cz1x} + F_{cz2x} } \right)f - \left( {F_{cz1y} - F_{cz2y} } \right){(}E{ + }P{)} - \sum\limits_{i = 1}^{4} {F_{dzix} } a \hfill \\ - F_{dz1y} (D + C) - \left( {F_{dz2y} - F_{dz3y} } \right)C + F_{dz4y} (D + C) - \left( {F_{sz1y} - F_{sz2y} } \right)E - \sum\limits_{i = 1}^{2} {F_{szix} } e = 0 \hfill \\ \end{gathered} \right. $$

The working state of the anchorage system is regarded as an ideal mechanical model. Due to the friction between the leg and the roadway floor, the static equation is written as a generalized absolute value equation.

8 $$ \begin{gathered} : \hfill \\ AX + B\left| X \right| = Y \hfill \\ \end{gathered} $$

wherein:

9 $$ A = \left[ {\begin{array}{*{20}c} { - \sin \lambda \cos \varepsilon } & { - \sin \lambda \cos \phi } & {\sin \gamma \cos \phi } & { - \sin \gamma \cos \varepsilon } & 0 & 0 \\ { - \sin \varepsilon } & { - \sin \phi } & { - \sin \phi } & { - \sin \varepsilon } & 0 & 0 \\ {\cos \lambda \cos \varepsilon } & {\cos \lambda \cos \phi } & {\cos \gamma \cos \phi } & {\cos \gamma \cos \varepsilon } & 0 & 0 \\ {D_{1} } & {D_{2} } & {D_{3} } & {D_{4} } & 0 & 0 \\ { - N\sin \lambda } & { - N\sin \lambda } & {N\sin \gamma } & { - N\sin \gamma } & 0 & 0 \\ { - B\sin \lambda - G\sin \varepsilon } & { - B\sin \lambda - G\sin \phi } & {B\sin \gamma + G\sin \phi } & { - B\sin \gamma + G\sin \phi } & 0 & 0 \\ \end{array} } \right] $$

10 $$ B = \left[ {\begin{array}{*{20}c} {\mu \cos \lambda } & { - \mu \cos \lambda } & { - \mu \cos \gamma } & {\mu \cos \gamma } & 0 & 0 \\ {\mu \cos \varepsilon } & {\mu \cos \phi } & {\mu \cos \phi } & {\mu \cos \varepsilon } & 0 & 0 \\ 0 & 0 & 0 & 0 & 0 & 0 \\ 0 & 0 & 0 & 0 & 0 & 0 \\ {N\mu \cos \lambda } & { - N\mu \cos \lambda } & { - N\mu \cos \gamma } & {N\mu \cos \gamma } & 0 & 0 \\ {D_{5} } & {D_{6} } & {D_{7} } & {D_{8} } & 0 & 0 \\ \end{array} } \right] $$

Wherein:

11 $$ \left\{ \begin{gathered} D_{1} = \sin \varepsilon N - \cos \lambda \cos \varepsilon B \hfill \\ D_{2} = \sin \phi N - \cos \lambda \cos \phi B \hfill \\ D_{3} = \cos \gamma \cos \phi B - \sin \phi N \hfill \\ D_{4} = \cos \gamma \cos \varepsilon B - \sin \varepsilon N \hfill \\ \end{gathered} \right.\;\;\;\;\;\left\{ \begin{gathered} D_{5} = B\mu \cos \lambda + G\mu \cos \varepsilon \hfill \\ D_{6} = B\mu \cos \lambda + G\mu \cos \phi \hfill \\ D_{7} = B\mu \cos \gamma + G\mu \cos \phi \hfill \\ D_{8} = - B\mu \cos \gamma + G\mu \cos \varepsilon \hfill \\ \end{gathered} \right. $$

12 $$ X = \left[ {\begin{array}{*{20}c} {F_{t1} } & {F_{t2} } & {F_{t3} } & {F_{t4} } & 0 & 0 \\ \end{array} } \right]^{{\text{T}}} $$

13 $$ Y = \left[ {\begin{array}{*{20}c} {y_{1} } & {y_{2} } & {y_{3} } & {y_{4} } & {y_{5} } & {y_{6} } \\ \end{array} } \right]^{{\text{T}}} $$

Wherein:

14 $$ y_{1} = - F_{cc1} + F_{cc2} - \left( {F_{cz1} - F_{cz2} } \right)\cos \alpha \cos \beta + \sum\limits_{i = 1}^{4} {F_{dzi} \cos \left( {\frac{\pi }{2} - \alpha } \right)} \cos \beta + \sum\limits_{i = 1}^{2} {F_{szi} } \cos \left( {\frac{\pi }{2} - \alpha } \right)\cos \beta $$

15 $$ y_{2} = \left( {F_{cz1} + F_{cz2} } \right)\cos \alpha \sin \beta + \sum\limits_{i = 1}^{4} {F_{dzi} \cos \left( {\frac{\pi }{2} - \alpha } \right)} \sin \beta + \sum\limits_{i = 1}^{2} {F_{szi} } \cos \left( {\frac{\pi }{2} - \alpha } \right)\sin \beta $$

16 $$ y_{3} = Mg + \left( {F_{cz1} + F_{cz2} } \right)\cos \alpha + \sum\limits_{i = 1}^{4} {F_{dzi} } \cos \left( {\frac{\pi }{2} - \alpha } \right) + \sum\limits_{i = 1}^{4} {F_{szi} } \cos \left( {\frac{\pi }{2} - \alpha } \right) + \sum\limits_{i = 1}^{4} {F_{dci} } + \sum\limits_{i = 1}^{4} {F_{sci} } $$

17 $$ \begin{aligned} y_{4} & = - Mgd + \left( {F_{cz1y} + F_{cz2y} } \right)H - \left( {F_{cz1z} + F_{cz2z} } \right)f + \sum\limits_{i = 1}^{4} {F_{dci} } b - \sum\limits_{i = 1}^{4} {F_{dziy} } k + \sum\limits_{i = 1}^{4} {F_{dziz} } a - \sum\limits_{i = 1}^{4} {F_{sci} } c \\ & \;\;\;\; - \sum\limits_{i = 1}^{4} {F_{sziy} } k - \sum\limits_{i = 1}^{4} {F_{sziz} } e \\ \end{aligned} $$

18 $$ \begin{aligned} y_{5} & = \left( {F_{cc1x} - F_{cc2} + F_{cz1} - F_{cz2x} } \right)H + \left( {F_{cz2z} - F_{cz1z} } \right)(P + E) - \sum\limits_{i = 1}^{4} {F_{dzix} } K - \left( {F_{dc1} + F_{dz1z} } \right)(D + C) \\ & \;\;\;\; - \left( {F_{dc2} + F_{dz2z} - F_{dc3} - F_{dz3z} } \right)C + \left( {F_{dc4} + F_{dz4z} } \right)(D + C) - \left( {F_{sc1} + F_{sz1z} - F_{sc2} - F_{sz2z} } \right)E - \sum\limits_{i = 1}^{2} {F_{szix} } K \\ \end{aligned} $$

19 $$ \begin{aligned} y_{6} & = \left( {F_{cc2} - F_{cc1} - F_{cz1x} + F_{cz2x} } \right)f + \left( {F_{cz1y} - F_{cz2y} } \right){(}E{ + }P{)} + \sum\limits_{i = 1}^{4} {F_{dzix} } a + F_{dz1y} (D + C) + \left( {F_{dz2y} - F_{dz3y} } \right)C \\ & \;\;\;\; - F_{dz4y} (D + C) + \left( {F_{sz1y} - F_{sz2y} } \right)E + \sum\limits_{i = 1}^{2} {F_{szix} } e \\ \end{aligned} $$

**Figure 5:**
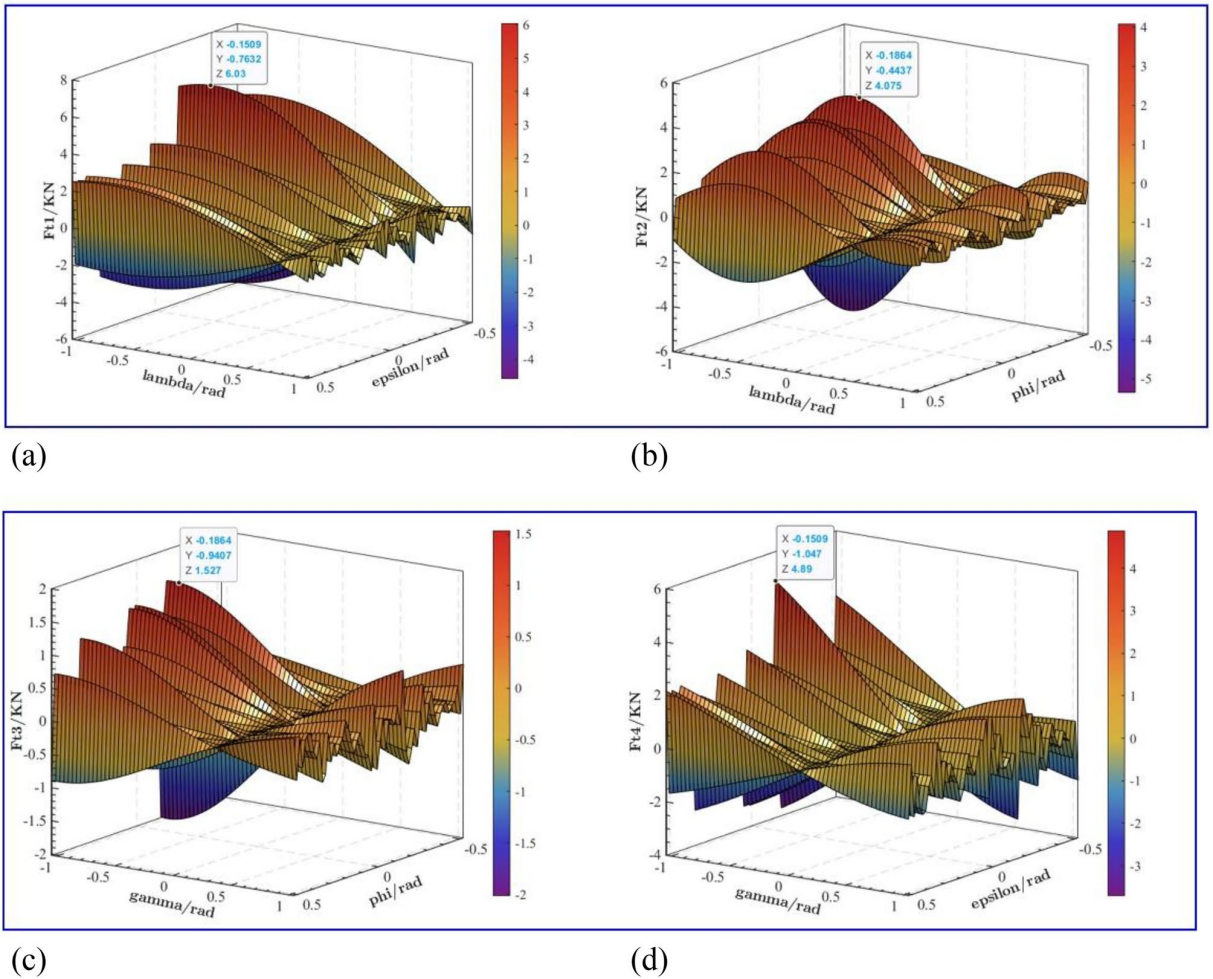
Diagram of support force changing with support angle (outward legs): (**a**) *F*_t1_ variation with Support Angle; (**b**) *F*_t2_ variation with Support Angle; (**c**) *F*_t3_ variation with Support Angle; (**d**) *F*_t4_ variation with Support Angle.

**Figure 6:**
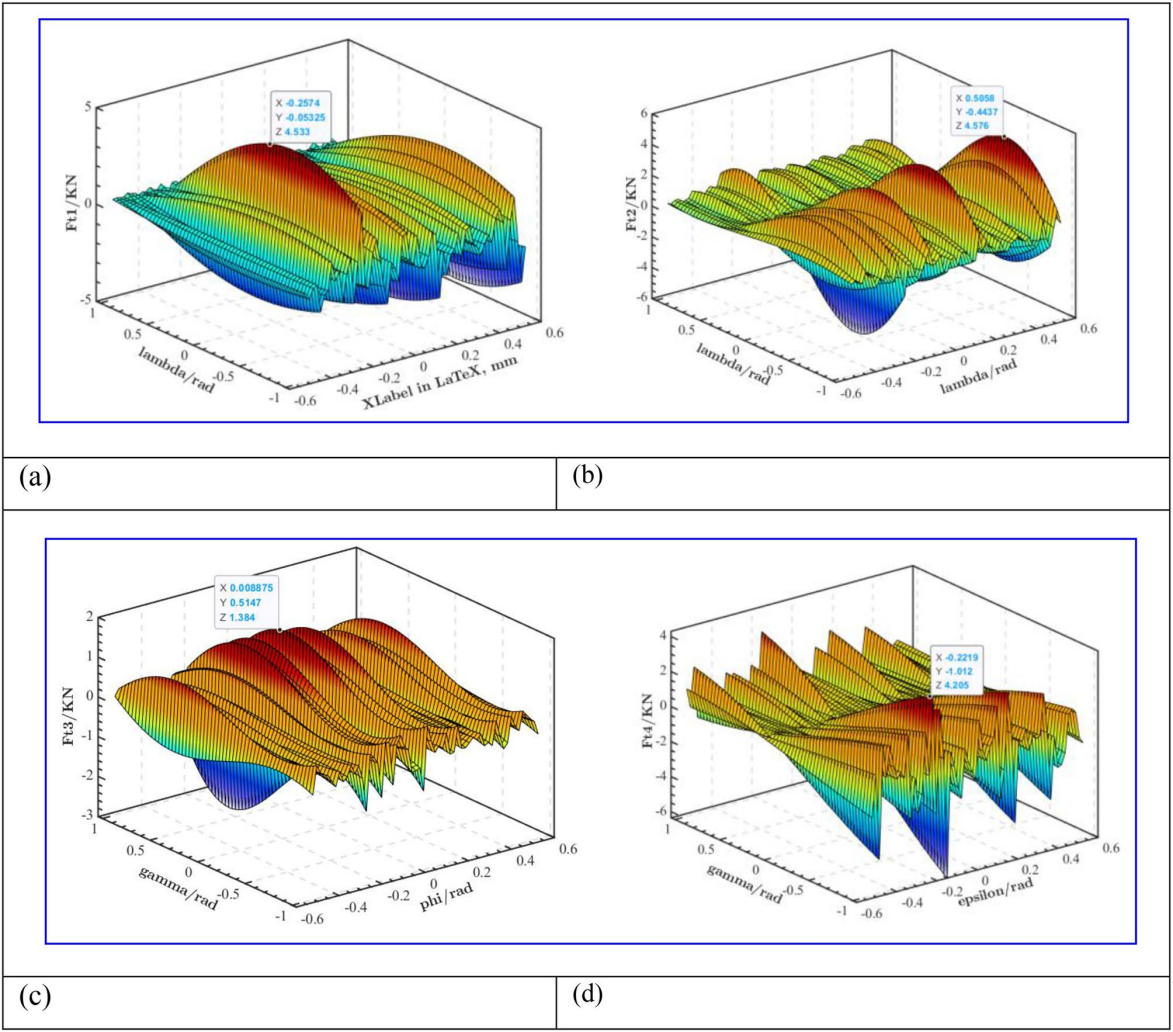
Diagram of support force changing with support angle (inward legs): (**a**) *F*_t1_ variation with Support Angle; (**b**) *F*_t2_ variation with Support Angle; (**c**) *F*_t3_ variation with Support Angle; (**d**) *F*_t4_ variation with Support Angle.

**Figure 7:**
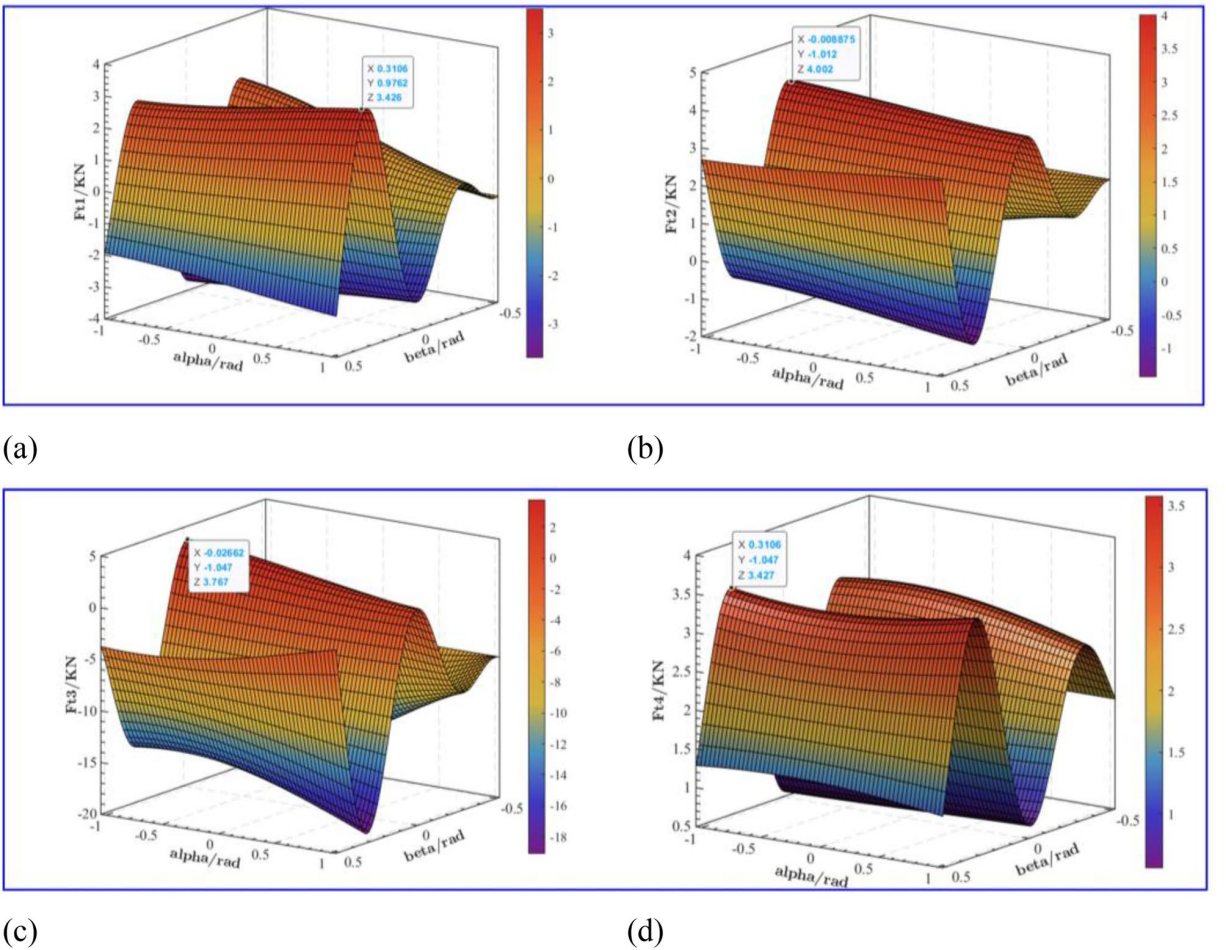
Support force changes with drilling angle: (**a**) *F*_t1_ variation with Drilling Angle; (**b**) *F*_t2_ variation with Drilling Angle; (**c**) *F*_t3_ variation with Drilling Angle; (**d**) *F*_t4_ variation with Drilling Angle.

Using Matlab numerical analysis software to solve the above equations, the change rule diagram of leg support force with support angle and drill pipe drilling angle can be obtained, as shown in Figs. 5, 6 and 7. Because the roadway floor is in a state of irregular deformation, the supporting force of the four legs is different. When the working condition of outrigger is the outward legs, as shown in Fig. 5. When the angle between leg 1 and the roadway floor is 0.15 rad and 0.76 rad respectively, the maximum supporting force of leg 1 is 6.03KN, as shown in Fig. 5a. When the angle between the support leg 2 and the roadway floor is 0.187 rad and 0.444 rad respectively, the maximum support force of support leg 2 is 4.075 KN, as shown in Fig. 5b; when the angle between leg 3 and the roadway floor is 0.186 rad and 0.941 rad respectively, the maximum support force of the leg 3 is 1.527 KN, as shown in Fig. 5c; When the angle between leg 4 and roadway floor is 0.151 rad and 1.047 rad, the maximum supporting force of leg 4 is 4.89 KN, as shown in Fig. 5d. The maximum support force of leg 1 is much larger than that of leg 3. It can be seen that the angle between the leg and the roadway floor has a great influence on the support force of the leg.

When the working condition of outrigger is the inward legs, as shown in Fig. 6. When the angle between leg 1 and the roadway floor is 0.257 rad and 0.053 rad respectively, the maximum supporting force of leg 1 is 4.533KN, as shown in Fig. 6a. When the angle between the support leg 2 and the roadway floor is 0.506 rad and 0.444 rad respectively, the maximum support force of support leg 2 is 4.576 KN, as shown in Fig. 6b; When the angle between leg 3 and the roadway floor is 0.009 rad and 0.515 rad respectively, the maximum support force of the leg 3 is 1.384 KN, as shown in Fig. 6c; When the angle between leg 4 and roadway floor is 0.222 rad and 1.012 rad, the maximum supporting force of leg 4 is 4.205 KN, as shown in Fig. 6d. Compared with the stress conditions of each leg in Fig. 5, the force changes of the inward legs and the outward legs are similar without obvious changes.

Due to the uneven condition of the roadway roof, the anchoring drilling force changes irregularly with the fluctuation of the roof, which has a certain influence on the supporting force of the leg. As shown in Fig. 7a, when the drilling incident angle $\alpha$ and $\beta$ are 0.311 rad and 0.976 rad respectively, the maximum supporting force of leg 1 is 3.426 KN; As shown in Fig. 7b, when the drilling incident angle $\alpha$ and $\beta$ are 0.009 rad and 1.012 rad respectively, the maximum supporting force of leg 2 is 4.002 KN ; As shown in Fig. 7c, when the drilling incident angle $\alpha$ and $\beta$ are 0.027 rad and 1.047 rad respectively, the maximum supporting force of leg 3 is 3.767 KN; As shown in Fig. 7d, when the drilling incident angle $\alpha$ and $\beta$ are 0.311 rad and 1.047 rad respectively, the maximum supporting force of the four legs is 3.427 KN. It can be seen that the drilling incident angle also has a great influence on the support force of the leg. The maximum support force is 4.002 KN according to the different angles of the leg and the different drilling angles. As the design criterion of the strength of the leg cylinder, this study has certain practical value.

## Construction of anchoring system dynamics model

Through the analysis of the whole structure of the anchorage system, it can be seen as a multi-mass and multi-attitude system, so the Lagrange method is used to establish the dynamic model of the anchorage group system. The complete Lagrangian equation can be generally expressed as Eq. (20).

20 $$ \frac{d}{dt}\left( {\frac{\partial T}{{\partial \dot{x}_{j} }}} \right) - \frac{\partial T}{{\partial x_{j} }} + \frac{\partial V}{{\partial x_{j} }} + \frac{\partial D}{{\partial \dot{x}_{j} }} = F_{j} (t) $$

wherein, *F*_*j*(*t*)_—external excitation force, $x_{j}$—generalized displacement, $\dot{x}_{j}$—generalized velocity, *T*—system kinetic energy, *V*—system potential energy, *D*—system energy dissipation function.

**Figure 8:**
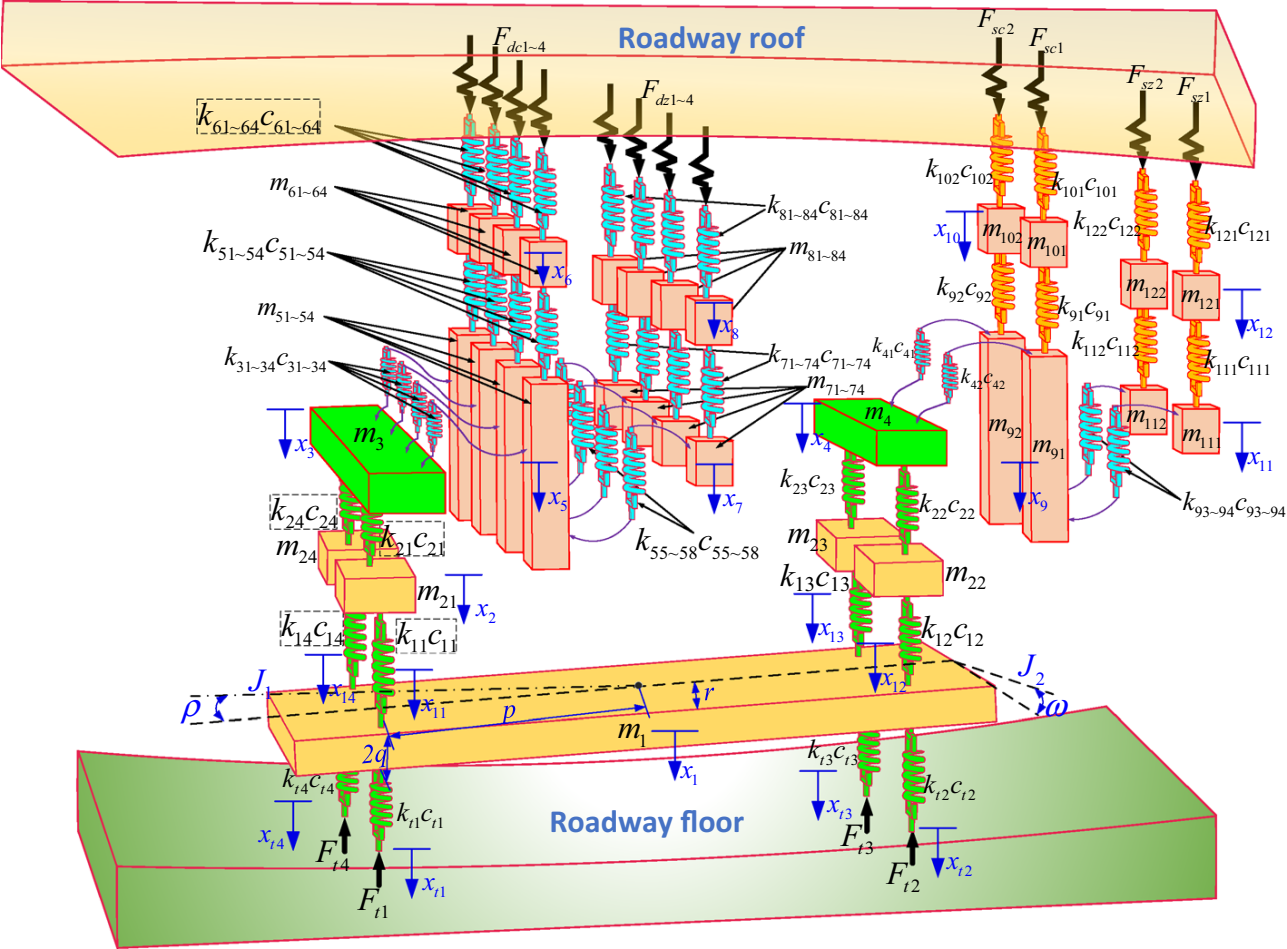
Construction of anchoring system dynamics mode.

The dynamic model of anchorage system is shown in Fig. 8. Wherein, *m*_1_, *m*_21~24_, *m*_3_, *m*_4_, *m*_51~54_, *m*_61~64_, *m*_71~74_, *m*_81~84_, *m*_91~92_, *m*_101~102_, *m*_111~112_, *m*_121~122_ represent the quality of the support platform, the quality of the front and rear support columns, the quality of the top anchor beam, the quality of the anchor cable beam, the quality of the four top anchor rig frame, the quality of the four top anchor rig support plate, the quality of the four top anchor rig power head, the quality of the four top anchor drill pipe, the quality of the two anchor rig frame, the quality of the two anchor support plate, the quality of the two anchor cable rig power head, the quality of the two anchor cable rig drill pipe.*k*_11~14_(*c*_11~14_), *k*_21~24_(*c*_21~24_), *k*_31~34_(*c*_31~34_), *k*_41~42_(*c*_41~42_), *k*_51~54_(*c*_51~54_), *k*_55~58_(*c*_55~58_), *k*_61~64_(*c*_61~64_), *k*_71~74_(*c*_71~74_), *k*_81~84_(*c*_81~84_), *k*_91~92_(*c*_91~92_), *k*_93~94_(*c*_93~94_), *k*_101~102_(*c*_101~102_), *k*_111~112_(*c*_111~112_), *k*_121~122_(*c*_121~122_) represents the equivalent stiffness (damping) between the support columns and the support platform, the support columns and the top anchor & anchor cable beam, the top anchor beam and the four top anchor frames, the anchor cable beam and the two anchor cable frames, the four top anchor frames and the four top anchor support plates, the four top anchor frames and the four top anchor power heads, the four top anchor support plates and the roadway roof, the four top anchor power heads and the four top anchor drill pipes, the four top anchor drill pipes and the roadway roof, the two anchor cable frames and the two anchor cable support plates, the two anchor cable frames and the two anchor cable power heads, the two anchor cable support plates and the roadway roof, the two anchor cable power heads and the two anchor cable drill pipes, the two anchor cable drill pipes and the roadway roof, respectively.*k*_t1~t4_(*c*_t1~t4_) represent the equivalent stiffness and equivalent damping between legs 1 ~ 4 and the ground respectively. *p* represents the distance between the center of gravity of the support platform and the leg. *q* is half the thickness of the support platform. *r* is half the width of the support platform. *J*_1_ represents the rotational inertia of the excavation direction of the support platform.$\rho$ represents the pitch vibration angle of the excavation direction of the support platform. *J*_2_ represents the horizontal rotational inertia of the supporting platform.$\varpi$ represents the horizontal vibration angle of the support platform. *F*_*dc*1~dc4_, *F*_*dz*1~dz4_, *F*_*sc*1~sc2_, and *F*_*sz*1~sz2_ represent the reaction force of four anchor support plates, the reaction force of four top anchor drilling, the reaction force of two anchor support plates and the reaction force of two anchor cable drilling respectively. *x*_1_ ~ *x*_12_ are expressed as the displacement of each mass block of the anchorage group along the longitudinal *x* direction.

Using the principle of energy method, the kinetic energy expression of the whole anchorage system can be obtained.

21 $$ \begin{aligned} T & = \frac{1}{2}m_{1} \dot{x}_{1}^{2} + \frac{1}{2}(m_{21} + m_{22} + m_{23} + m_{24} )\dot{x}_{2}^{2} + \frac{1}{2}m_{3} \dot{x}_{3}^{2} + \frac{1}{2}m_{4} \dot{x}_{4}^{2} + \frac{1}{2}\sum\limits_{i = 1}^{4} {m_{5i} } \dot{x}_{5}^{2} + \frac{1}{2}\sum\limits_{i = 1}^{4} {m_{6i} } \dot{x}_{6}^{2} \\ & \;\;\; + \frac{1}{2}\sum\limits_{i = 1}^{4} {m_{7i} } \dot{x}_{7}^{2} + \frac{1}{2}\sum\limits_{i = 1}^{4} {m_{8i} } \dot{x}_{8}^{2} + \frac{1}{2}\sum\limits_{i = 1}^{2} {m_{9i} } \dot{x}_{9}^{2} + \frac{1}{2}\sum\limits_{i = 1}^{2} {m_{10i} } \dot{x}_{10}^{2} + \frac{1}{2}\sum\limits_{i = 1}^{2} {m_{11i} } \dot{x}_{11}^{2} + \frac{1}{2}\sum\limits_{i = 1}^{2} {m_{12i} } \dot{x}_{12}^{2} \\ & \;\;\; + \frac{1}{2}J_{1} \dot{\rho }^{2} + \frac{1}{2}J_{2} \dot{\varpi }^{2} \\ \end{aligned} $$

wherein,$$m_{2i} = m_{2} (i = 1\sim 4)$$,$$m_{2i} = m_{2} (i = 1\sim 4)$$,$$m_{3i} = m_{3} (i = 1\sim 4)$$,$$m_{5i} = m_{5} (i = 1\sim 4)$$, $$m_{6i} = m_{6} (i = 1\sim 4)$$,$$m_{7i} = m_{7} (i = 1\sim 4)$$,$$m_{8i} = m_{8} (i = 1\sim 4)$$,$$m_{9i} = m_{9} (i = 1\sim 2)$$,$$m_{10i} = m_{10} (i = 1\sim 2)$$,$$m_{11i} = m_{11} (i = 1\sim 2)$$,$$m_{12i} = m_{12} (i = 1\sim 2)$$.

This kinetic energy expression can be simplified as Eq. (22).

22 $$ \begin{aligned} T & = \frac{1}{2}m_{1} \dot{x}_{1}^{2} + 2m_{2} \dot{x}_{2}^{2} + \frac{1}{2}m_{3} \dot{x}_{3}^{2} + \frac{1}{2}m_{4} \dot{x}_{4}^{2} + 2m_{5} \dot{x}_{5}^{2} + 2m_{6} \dot{x}_{6}^{2} + 2m_{7} \dot{x}_{7}^{2} \\ & \;\;\; + 2m_{8} \dot{x}_{8}^{2} + m_{9} \dot{x}_{9}^{2} + m_{10} \dot{x}_{10}^{2} + m_{11} \dot{x}_{11}^{2} + m_{12} \dot{x}_{12}^{2} + \frac{1}{2}J_{1} \dot{\rho }^{2} + \frac{1}{2}J_{2} \dot{\varpi }^{2} \\ \end{aligned} $$

wherein, $\dot{x}_{i} (i = 1\sim 12)$—Mass block velocity.

Since the body swing vibration is small, it can be assumed that $\sin \rho \approx \rho$,$\sin \varpi \approx \varpi$. The following Eq. (23) can be obtained.

23 $$ \left\{ \begin{gathered} x_{t1} = x_{1} - l_{1} \rho - l_{2} \varpi \hfill \\ x_{t2} = x_{1} + l_{1} \rho - l_{2} \varpi \hfill \\ x_{t3} = x_{1} + l_{1} \rho + l_{2} \varpi \hfill \\ x_{t4} = x_{1} - l_{1} \rho + l_{2} \varpi \hfill \\ \end{gathered} \right.\;\;\;\;\;\left\{ \begin{gathered} x_{11} = x_{1} + l_{1} \rho + l_{2} \varpi \hfill \\ x_{12} = x_{1} - l_{1} \rho + l_{2} \varpi \hfill \\ x_{13} = x_{1} - l_{1} \rho - l_{2} \varpi \hfill \\ x_{14} = x_{1} + l_{1} \rho - l_{2} \varpi \hfill \\ \end{gathered} \right. $$

The potential energy of the anchorage system can be expressed as Eq. (24).

24 $$ \begin{aligned} V & = \frac{1}{2}k_{t1} x_{t1}^{2} + \frac{1}{2}k_{t2} x_{t2}^{2} + \frac{1}{2}k_{t3} x_{t3}^{2} + \frac{1}{2}k_{t4} x_{t4}^{2} + \frac{1}{2}k_{11} (x_{11} - x_{2} )^{2} + \frac{1}{2}k_{12} (x_{12} - x_{2} )^{2} + \frac{1}{2}k_{13} (x_{13} - x_{2} )^{2} \\ & \;\;\; + \frac{1}{2}k_{14} (x_{14} - x_{2} )^{2} + \frac{1}{2}(k_{21} + k_{24} )(x_{2} - x_{3} )^{2} + \frac{1}{2}(k_{22} + k_{23} )(x_{2} - x_{4} )^{2} + \frac{1}{2}\sum\limits_{i = 1}^{4} {k_{3i} } (x_{3} - x_{5} )^{2} \\ & \;\;\; + \frac{1}{2}\sum\limits_{i = 1}^{4} {k_{5i} } (x_{5} - x_{6} )^{2} + \frac{1}{2}\sum\limits_{i = 1}^{4} {k_{6i} } x_{6}^{2} + \frac{1}{2}\sum\limits_{i = 5}^{8} {k_{5i} } (x_{5} - x_{7} )^{2} + \frac{1}{2}\sum\limits_{i = 1}^{4} {k_{7i} } (x_{7} - x_{8} )^{2} + \frac{1}{2}\sum\limits_{i = 1}^{4} {k_{8i} } x_{8}^{2} \\ & \;\;\; + \frac{1}{2}(k_{41} + k_{42} )(x_{4} - x_{9} )^{2} + \frac{1}{2}(k_{91} + k_{92} )(x_{9} - x_{10} )^{2} + \frac{1}{2}(k_{93} + k_{94} )(x_{9} - x_{11} )^{2} \\ & \;\;\; + \frac{1}{2}(k_{111} + k_{112} )(x_{11} - x_{12} )^{2} + \frac{1}{2}(k_{121} + k_{122} )x_{12}^{2} \\ \end{aligned} $$

wherein, $$l_{1} = \sqrt {p^{2} + q^{2} }$$,$$l_{2} = \sqrt {q^{2} + r^{2} }$$.

The six drilling rigs have the same structural form and symmetrical layout. For this reason, it can be assumed that the stiffness of some similar parts is approximately equal.

$$ \begin{gathered} k_{ti} = k_{t} (i = 1\sim 4),k_{1i} = k_{1} (i = 1\sim 4),k_{2i} = k_{2} (i = 1\sim 4),k_{3i} = k_{3} (i = 1\sim 4),k_{5i} = k_{5} (i = 1\sim 4),k_{6i} = k_{6} (i = 1\sim 4), \hfill \\ k_{5i} = k_{5/2} (i = 5\sim 8),k_{7i} = k_{7} (i = 1\sim 4),k_{8i} = k_{8} (i = 1\sim 4),k_{41} = k_{42} = k_{4} ,k_{91} = k_{92} = k_{9/1} , \hfill \\ k_{93} = k_{94} = k_{9/2} ,k_{111} = k_{112} = k_{11} ,k_{121} = k_{122} = k_{12} \hfill \\ \end{gathered} $$

The potential energy expression for this anchorage system can be simplified as Equation ( 25 ).

25 $$ \begin{gathered} V = 2k_{t} \left\{ {(x_{1} - l_{1} \rho - l_{2} \varpi )^{2} + (x_{1} + l_{1} \rho - l_{2} \varpi )^{2} + (x_{1} + l_{1} \rho + l_{2} \varpi )^{2} + (x_{1} - l_{1} \rho + l_{2} \varpi )^{2} } \right\} \\ + \frac{1}{2}k_{1} \left\{ \begin{gathered} (x_{1} + l_{1} \rho + l_{2} \varpi - x_{2} )^{2} + (x_{1} - l_{1} \rho + l_{2} \varpi - x_{2} )^{2} + (x_{1} - l_{1} \rho - l_{2} \varpi - x_{2} )^{2} \hfill \\ + (x_{1} + l_{1} \rho - l_{2} \varpi - x_{2} )^{2} \hfill \\ \end{gathered} \right\} \\ + k_{2} (x_{2} - x_{3} )^{2} + k_{2} (x_{2} - x_{4} )^{2} + 2k_{3} (x_{3} - x_{5} )^{2} + 2k_{5/1} (x_{5} - x_{6} )^{2} + 2k_{6} x_{6}^{2} \\ + 2k_{5/2} (x_{5} - x_{7} )^{2} + 2k_{7} (x_{7} - x_{8} )^{2} + 2k_{8} x_{8}^{2} + k_{4} (x_{4} - x_{9} )^{2} + k_{9/1} (x_{9} - x_{10} )^{2} \\ + k_{9/2} (x_{9} - x_{11} )^{2} + k_{11} (x_{11} - x_{12} )^{2} + k_{12} x_{12}^{2} \\ \end{gathered} $$

The damping values in the energy dissipation equation are simplified by the above method, and the energy dissipation expression (26) of the anchorage system can be obtained.

26 $$ \begin{aligned} D & = 2c_{t} \left\{ {(\dot{x}_{1} - l_{1} \dot{\rho } - l_{2} \dot{\varpi })^{2} + (\dot{x}_{1} + l_{1} \dot{\rho } - l\dot{\varpi })^{2} + (\dot{x}_{1} + l_{1} \dot{\rho } + l\dot{\varpi })^{2} + (\dot{x}_{1} - l_{1} \dot{\rho } + l\dot{\varpi })^{2} } \right\} \\ & \;\;\; + \frac{1}{2}c_{1} \left\{ \begin{gathered} (\dot{x}_{1} + l_{1} \dot{\rho } + l_{2} \dot{\varpi } - \dot{x}_{2} )^{2} + (\dot{x}_{1} - l_{1} \dot{\rho } + l_{2} \dot{\varpi } - \dot{x}_{2} )^{2} + (\dot{x}_{1} - l_{1} \dot{\rho } - l_{2} \dot{\varpi } - \dot{x}_{2} )^{2} \hfill \\ + (\dot{x}_{1} + l_{1} \dot{\rho } - l_{2} \dot{\varpi } - \dot{x}_{2} )^{2} \hfill \\ \end{gathered} \right\} \\ & \;\;\; + c_{2} (\dot{x}_{2} - \dot{x}_{3} )^{2} + c_{2} (\dot{x}_{2} - \dot{x}_{4} )^{2} + 2c_{3} (\dot{x}_{3} - \dot{x}_{5} )^{2} + 2c_{5/1} (\dot{x}_{5} - \dot{x}_{6} )^{2} + 2c_{6} \dot{x}_{6}^{2} \\ & \;\;\; + 2c_{5/2} (\dot{x}_{5} - \dot{x}_{7} )^{2} + 2c_{7} (\dot{x}_{7} - \dot{x}_{8} )^{2} + 2c_{8} \dot{x}_{8}^{2} + c_{4} (\dot{x}_{4} - \dot{x}_{9} )^{2} + c_{9/1} (\dot{x}_{9} - \dot{x}_{10} )^{2} \\ & \;\;\; + c_{9/2} (\dot{x}_{9} - \dot{x}_{11} )^{2} + c_{11} (\dot{x}_{11} - \dot{x}_{12} )^{2} + c_{12} \dot{x}_{12}^{2} \\ \end{aligned} $$

## Dynamic simulation analysis of anchorage system

### Vibration response analysis of key components

**Figure 9:**
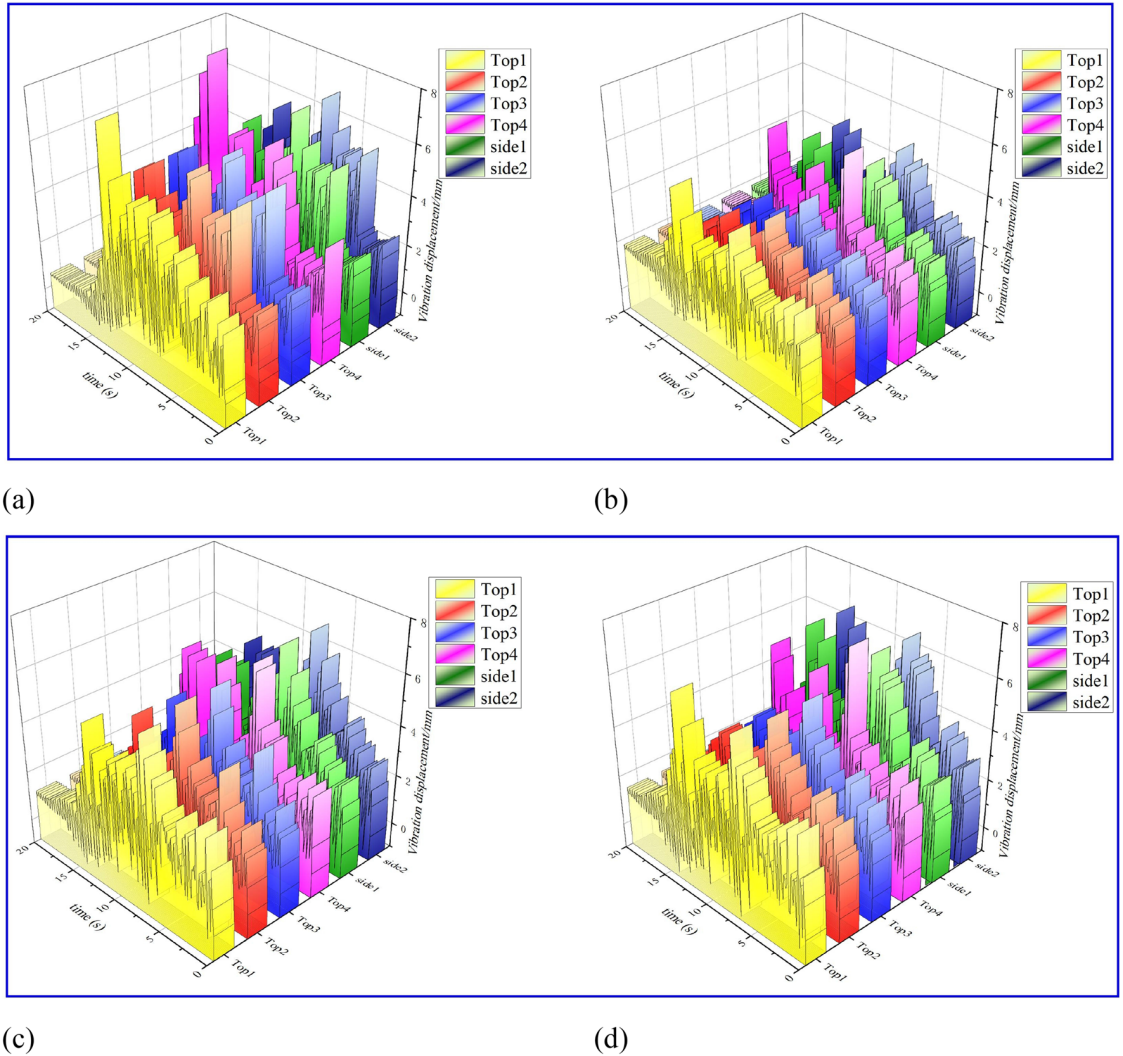
Vibration displacement response diagram of each drilling unit module: (**a**) vibration displacement diagram of drill pipe of different rigs; (**b**) vibration displacement diagram of different drill support plates; (**c**) vibration displacement diagram of power heads of different rigs; (**d**) vibration displacement diagram of different drilling rigs.

In the process of anchoring drilling, factors such as uneven roof and floor of roadway, different drilling and supporting angles, and variable load characteristics of drilling are considered. This section analyzes the maximum vibration response condition of the actual anchoring drilling system (4 top anchors + 2 side anchors working at the same time) and obtains the vibration response law of each mass block of the following anchoring group system. Since the action time of external load is 0–14 s, the response of each component tends to be stable after 14 s, which is consistent with the actual drilling process. As shown in Fig. 9a, the overall vibration change trend of the drill pipe of the six drilling rigs is similar. The top anchor 1 # and the top anchor 4 # reach the maximum peak of 7.5 mm around 14 s, and the rest of the drilling rigs reach the maximum peak of 6.35 mm around 10 s. Because the top anchor 1 # and the top anchor4 # are in the cantilever state, the response time of the disturbance during the drilling process is prolonged, and the vibration response is large, which is in line with the actual situation. As shown in Fig. 9b, the drilling rig support plate is weak compared with the drill pipe due to the fact that it only bears the auxiliary support effect and the coupling effect of coal and rock. Therefore, the overall peak value of each support plate is lower than that of the drill pipe. The maximum peak value is still generated in the top anchor 1 # and the top anchor 4 #, and the peak value is 6 mm. The vibration peak of other drilling rig support plates is generated in 7 s, and the maximum peak value is 5.8 mm.

As shown in Fig. 9c, d, the peak value of the vibration response displacement of the power head and the rig frame of the top anchor 4# in the six rigs is the largest, and the maximum peak value of the vibration is about 7.5 mm around 7 s. The maximum peak value of the power head and rig frame of other rigs is about 6 mm; Considering the uneven working conditions of the roof and floor and the instability of the cantilever beam, the angle of the top anchor 4# drilling hole changes, and the large vibration of the drilling rig frame leads to the above results, which is in line with the actual drilling operation conditions.

**Figure 10:**
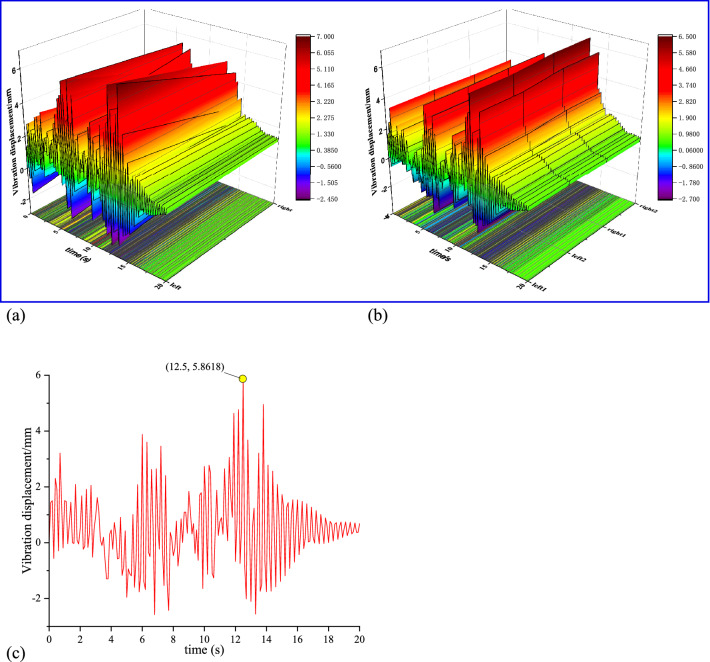
Vibration response diagram of main components of anchoring system: (**a**) vibration displacement diagram of top anchor and anchor cable beam; (**b**) vibration displacement diagram of front and rear support column beam; (**c**) vibration displacement diagram of support platform.

The top anchor beam is mainly used as the supporting part of the four top anchor drilling rigs, and the anchor cable beam is used as the connecting part of the two anchor cable drilling rigs. As shown in Fig. 10a, the top anchor beam reaches the maximum peak of 7 mm at about 14 s, and the anchor cable beam reaches the maximum peak of about 5 mm at about 12 s. Considering the different stress states of the two beams during drilling, the simulation results are in line with the actual state. Similarly, the bearing load of the supporting column changes due to the different number of supporting rigs. Although the top anchor beam has a large disturbance under the condition of four rigs drilling at the same time, considering the support plate of the rig under the support condition, other components of the anchoring system constitute the ' upper and lower support ' type stable structure. Therefore, the vibration response value of the anchor beam is larger than that of the anchor beam, as shown in Fig. 10b. As the main load-bearing component of the system, the support platform reaches the maximum vibration displacement peak of 5.86 mm under the drilling time of 12.5 s during the simultaneous drilling of multiple rigs. As shown in Fig. 10c, the vibration response peak of other components is slightly smaller, which is in line with the actual working conditions.

### Comparative analysis of drill pipe characteristics under cantilever extension and retraction operation

**Figure 11:**
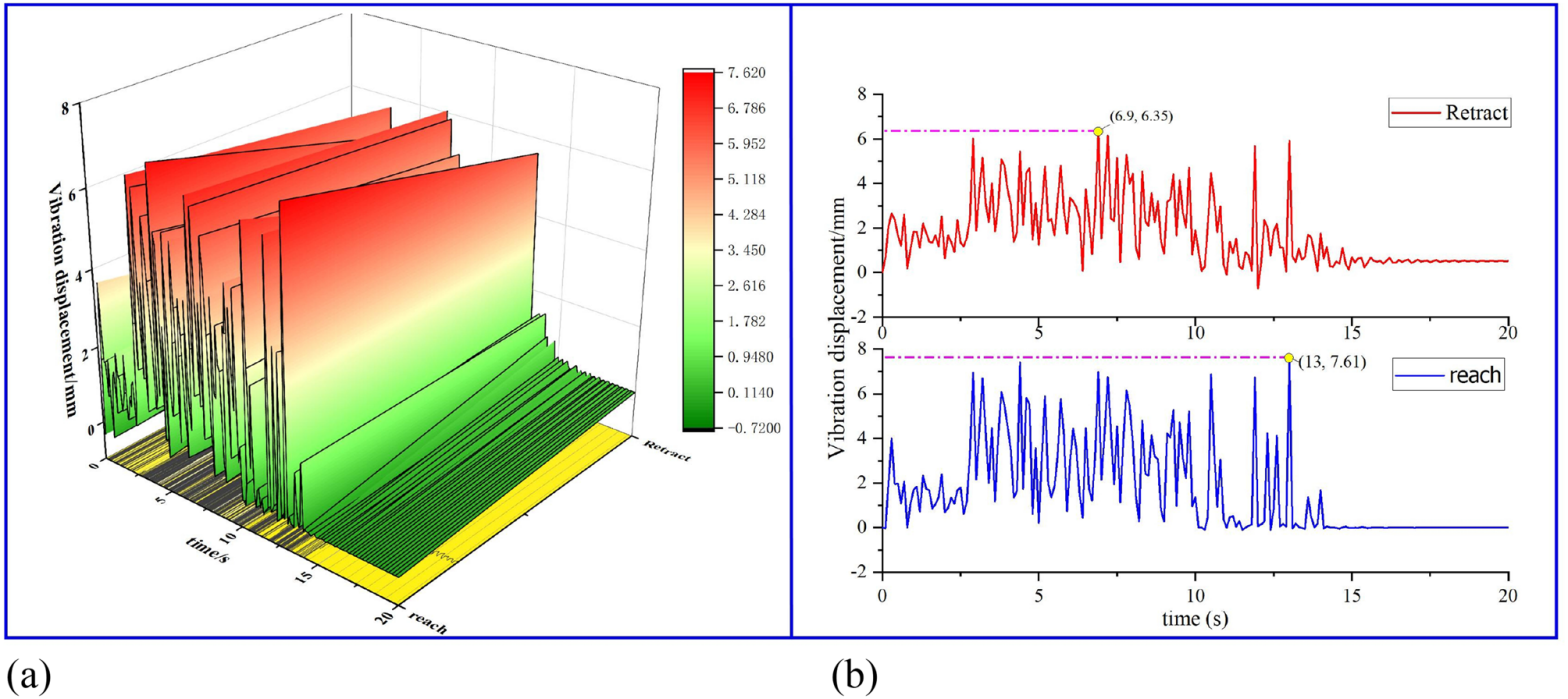
Vibration displacement diagram of drill pipe under cantilever extension or retraction operation: (**a**) vibration displacement surface diagram of drill pipe during cantilever extension/retraction operation; (**b**) projection of vibration displacement of drill pipe during cantilever extension/retraction operation.

Considering the technological process requirements of the anchoring system, there is a lateral extension drilling condition in the top anchor drilling opportunity. The cantilever extension state and the full retraction state will have a certain impact on the stability of the system. Therefore, for the two states of lateral top anchor drilling rig extension and retraction, the intermediate reference drilling rig is simulated. The simulation results are shown in Fig. 11. In the retracted state of both sides of the drilling rig, the intermediate reference drilling rig reaches the maximum vibration peak of 6.35 mm at 6.9 s; In the extended state on both sides of the rig, the middle benchmark rig reaches the maximum vibration peak 7.61 mm at 13 s; Through the above analysis, it can be seen that the drilling rigs on both sides have a greater impact on the drilling stability of the reference drilling rig under the extended state than the retracted state, and the drilling effect is relatively poor.

### Comparative snalysis of drill pipe vibration characteristics under single and multiple drilling rigs

Considering the requirements of the anchoring parameters of the roadway section, there are multiple drilling rig drilling operations under different working conditions. The specific working conditions are as follows.

Working condition 1 #: 2 top anchor drilling rigs work at the same time.

Condition 2 #: 4 top anchor drilling rigs working at the same time.

Working condition 3 #: 2 top anchor + 4 anchor cable drilling rigs working at the same time.

Condition 4 #: 2 top anchors + 2 anchor rigs working at the same time.

**Figure 12:**
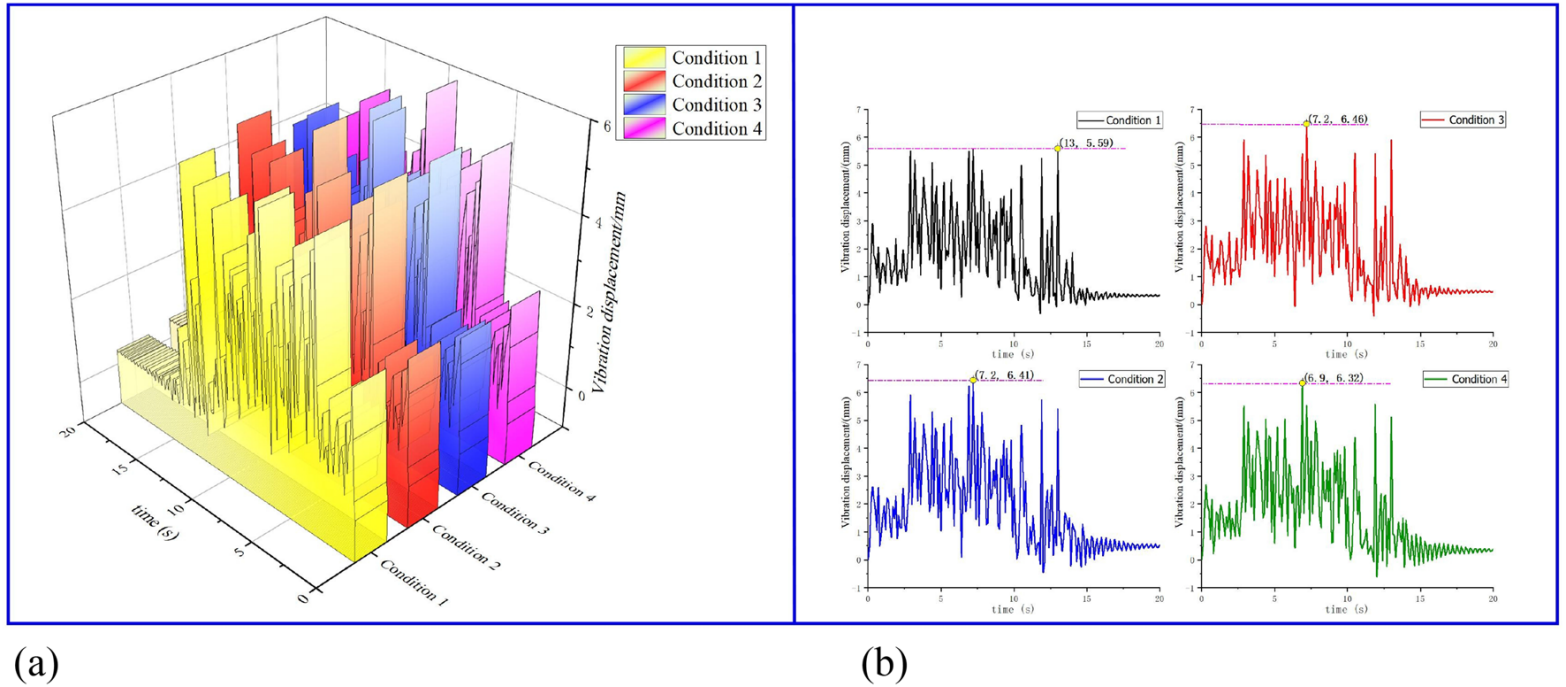
Vibration displacement diagram of drill pipe under multi-driller combination drilling: (**a**) curved surface of vibration displacement of drill pipe in multi drill combination drilling; (**b**) projection of vibration displacement of drill pipe in multi rig combination drilling.

As shown in Fig. 12, under the condition of 1 # ~ 4 #, the vibration displacement peaks of the benchmark drilling rig are 5.59 mm, 6.46 mm, 6.41 mm, and 6.32 mm, respectively. Among them, under the condition of 2 # and 3 #, the vibration peak of the benchmark drilling rig is the largest and close to each other. Under the condition of working condition 1 #, only the top anchoring drilling operation is carried out without other factors, so the vibration peak of the drill pipe of the benchmark rig is the smallest, which is in line with the actual working condition.

### Analysis of drill pipe vibration characteristics under different drilling incidence angles

**Figure 13:**
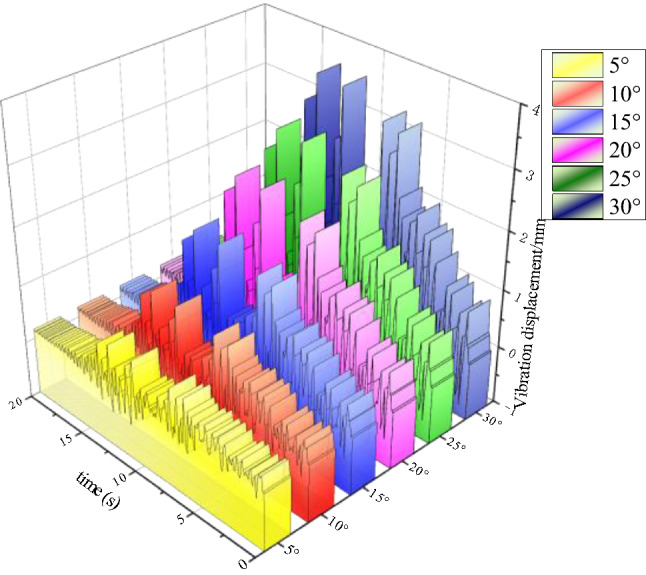
Vibration displacement diagram of drill pipe under different drilling incident angles.

Considering that the drilling angle changes due to the uneven roof during the anchoring drilling process, different drilling incident angles have different effects on the stability of drilling, drilling effect, and drill pipe stress. As shown in Fig. 13, with the gradual increase of the drilling incident angle, the peak vibration of the drill pipe of the reference rig increases in turn, and the drilling stability is worse. When the drilling angle is 30°, the maximum peak value is 3.5 mm, which meets the actual drilling conditions.

## Experimental verification

**Figure 14:**
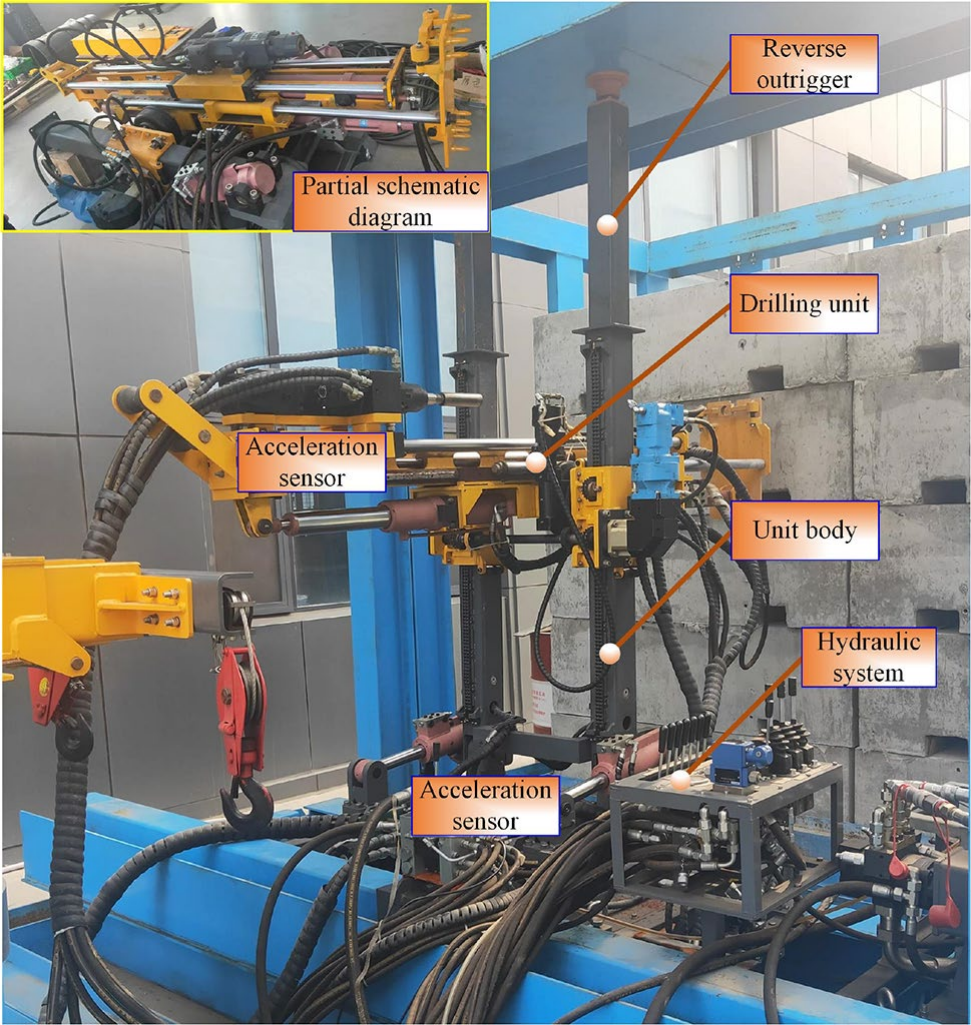
Anchorage system test prototype.

In order to obtain the vibration characteristic mechanism of the anchorage group system, the experimental prototype of the anchorage system is designed. The anchoring drilling test is carried out by simulating the field coal rock properties, as shown in Fig. 14. To detect the vibration characteristics of the anchoring system during drilling, the B3X23S10 piezoelectric acceleration sensor (IEPE) and the BA9004 portable dynamic signal analyzer were used to detect the vibration characteristics of the drilling system. The software of the analysis system can realize the vibration response analysis of the support platform and the drilling frame during the drilling process.

**Figure 15:**
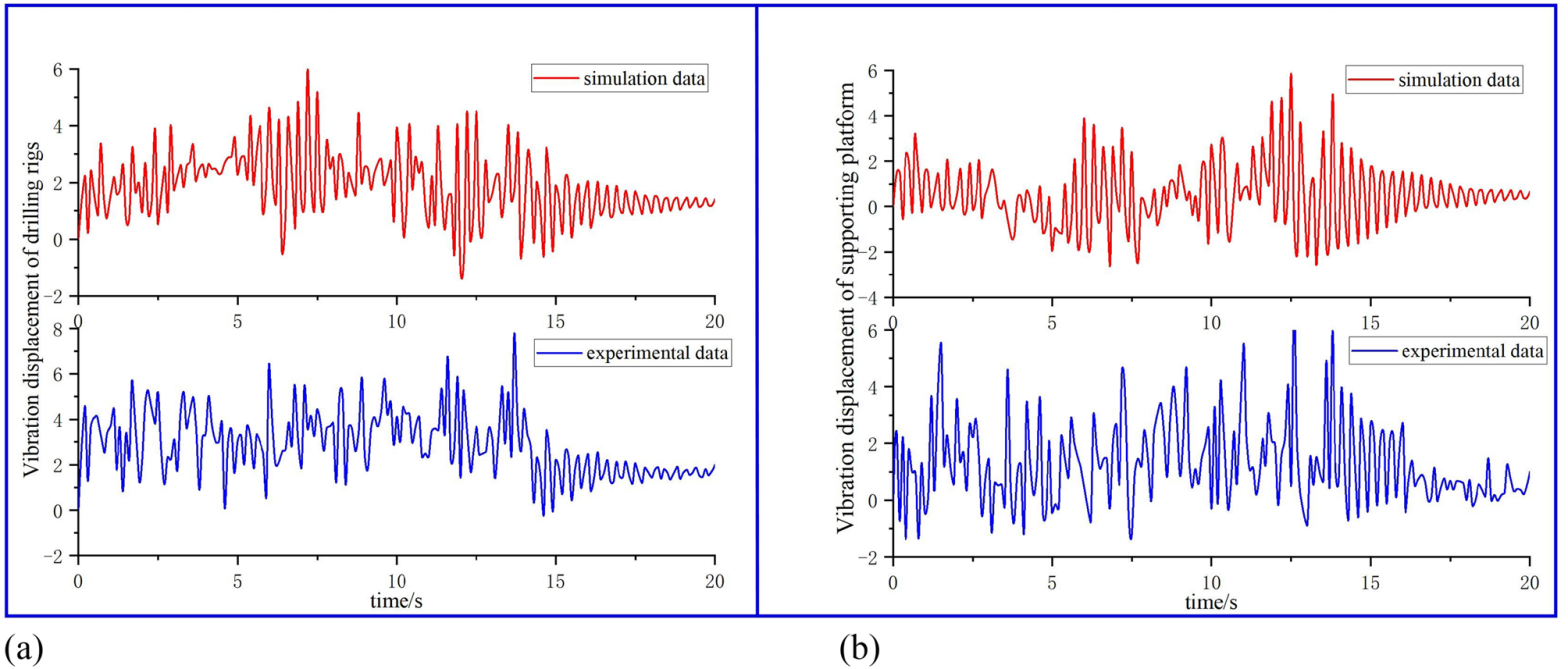
Vibration simulation and test comparison diagram of key components of anchoring system: (**a**) comparison diagram of vibration response theory and test of drilling rigs; (**b**) comparison diagram of vibration response theory and test of supported platform.

As shown in Fig. 15a and b, the maximum vibration amplitudes of the drilling rig frame and the supporting platform during the actual drilling operation are 7.5 mm and 6 mm, respectively. After the 14 s drilling rig stops working, the vibration displacements of both are gradually reduced, which is similar to the theoretical vibration amplitude and vibration law. The test results can verify the accuracy of the above vibration simulation results. The research in this paper can provide a theoretical basis for the vibration reduction of key components in the drilling process of the anchoring system and improve the stability of anchoring drilling.

## Conclusions

1. Considering the unevenness of the roof and floor of the coal mine roadway, the static characteristics of the anchoring system are analyzed under the condition of multiple drilling rigs drilling at the same time. The results show that the maximum support force is 4.002 KN when the leg angle is different and the drilling angle is different. As the design criterion of the strength of the leg cylinder, this study has certain practical value.
2. By constructing the coupling vibration model of the multi-drilling rig drilling process, the Lagrange method is used to solve the vibration law of key components of the anchorage system. The results show that:
  1. The vibration of the drill pipe in the cantilever extension state is more severe than that in the retracted state, and the maximum vibration peak reaches 7.61 mm.
  2. The vibration response of the drill pipe is the most intense under the condition of four top anchor drilling rigs drilling at the same time. Under the working condition of only two drilling rigs, the vibration response of the drill pipe is the smallest.
  3. As the drilling angle of the drilling rig increases, the vibration response of the drill pipe is more severe and the vibration amplitude is larger.
3. A test prototype is built to simulate the actual anchoring drilling process, and the vibration law of the support platform and the drilling rig is obtained through the vibration detection system. The test results show that the vibration law of the key components is approximately the same as the theoretical simulation results. The relevant theoretical results can provide a technical basis for the drilling stability of the anchoring system.
4. There is a certain difference between the mechanical model constructed in this paper and the on-site anchoring drilling process, which is mainly reflected in the fact that the excavating process will cause a certain disturbance to the roadway roof, which will be transmitted to the anchoring system. In the future, the coupling dynamic characteristics between the roadheader, the anchoring system, and the roof can be analyzed to make it more suitable for the actual anchoring operation.

## Data Availability

All data generated or analysed during this study are included in this published article.
